# Draft Genome Sequence of *Aeromonas dhakensis* Strain F2S2-1, Isolated from the Skin Surface of an Indian Oil Sardine (*Sardinella longiceps*)

**DOI:** 10.1128/genomeA.00494-16

**Published:** 2016-08-18

**Authors:** Mohan Nadiga, V. V. Vaidyanathan, Thangavelu Thayumanavan

**Affiliations:** a Syngene International Ltd., Bangalore, India; b Dr G R Damodaran College of Science, Coimbatore, India

## Abstract

Draft genome sequencing of *Aeromonas dhakensis* strain F2S2-1, isolated from the skin surface of an Indian oil sardine (*Sardinella* longiceps), has been carried out. The draft genome was roughly 4.7 Mb in size with 61.7% G+C content. Annotation of the genome yielded 4,337 genes coding for proteins, tRNAs, and rRNAs. Annotation also revealed the presence of 52 genes linked to resistance to antibiotics/toxic compounds. Pathway analysis revealed the presence of novobiocin biosynthetic genes and genes for biosynthesis of a siderophore group on nonsynthetic peptides.

## GENOME ANNOUNCEMENT

*Aeromonas hydrophila* is a well-known aquatic bacterium, widely distributed in marine ecosystems across the world (1). It is considered an opportunistic pathogen and causes infections in humans, fish, frog, and other animals (2, 3). *A. hydrophila* is widely prevalent in sea food sold in India and was identified as causative agent in diarrheal and other gastrointestinal tract infections in children (1, 2).

The complete and draft genome sequences of *A. hydrophila* were recently reported (4, 5). In South Asian countries such as India and Bangladesh, *A. hydrophila* strains exhibit antibiotic resistance, thus assuming clinical significance (6). Therefore, understanding the genome sequence of *A. hydrophila* strains prevalent in developing countries is important for diagnostic and treatment purposes. Here we report the draft genome sequence of *A. dhakensis* strain F2S2-1 isolated from skin of Indian Sardine (*Sardinella longiceps*), a common sea fish that is an important source of protein for the population.

*A. dhakensis* F2S2-1 was isolated from the external skin surface of an Indian sardine by standard culturing methods. Genomic DNA was extracted using a Gentra genomic DNA isolation kit (Qiagen). Genomic sequencing was performed on an Illumina Nextseq500 (2- × 150-bp chemistry). After quality trimming (score of >Q30), a total of ~2.1 million reads were assembled using SPAdes (version 3.6.0) software (7). Assembled contigs were further extended using the SSPACE program (8). *De novo* assembly resulted in 87 contigs, which represents ~4.7 mb of the draft genome (*N*_50_ 260,485 bp, maximum contig length 727,174 bp, and G+C content ~61.7%). The genome sequence of *Aeromonas dhakensis* F2S2-1 is in good agreement with the published sequences for *A. hydrophila* genomes (4.5 to 5.0 Mb).

Annotation of the genome using RAST (9) identified 4,337 genes, which included 4,224 protein-coding, 89 tRNA, and 24 rRNA genes. RAST predicted numerous genes encoding virulence and defense factors, of which 52 are related to resistance to antibiotics/toxic compounds. These include10 genes for multidrug resistance efﬂux pumps, 5 for the type I secretion system, 18 for the type IV secretion system, 10 for hemolysin and hemolysin-like genes, 3 for β-lactamases, 1 for a multiple antibiotic resistance locus, 1 gene for a lysozyme inhibitor, and 4 genes encoding ﬂuoroquinolone resistance.

Pathway analysis (10) revealed the presence of 4 novobiocin biosynthesis genes (*hisC, aspC, tyrA*, and *tyrB*) and 5 genes (*entA, entB, entC, entE*, and *menF*) involved in biosynthesis of a siderophore group of nonribosomal peptides.

### Accession number(s).

This whole-genome shotgun project has been deposited at DDBJ/ENA/GenBank under the accession number LZFM00000000. The version described in this paper is version LZFM01000000.
